# Single versus bilateral lung transplantation in idiopathic pulmonary fibrosis: A systematic review and meta-analysis

**DOI:** 10.1371/journal.pone.0233732

**Published:** 2020-05-21

**Authors:** Diandian Li, Yi Liu, Bo Wang

**Affiliations:** 1 Department of Respiratory and Critical Care Medicine, West China Hospital of Sichuan University, Chengdu, Sichuan, China; 2 West China School of Medicine, Sichuan University, Chengdu, Sichuan, China; Toranomon Hospital, JAPAN

## Abstract

**Objective:**

Lung transplantation remains the only curative treatment for end-stage lung disease, conferring a better survival for some IPF patients, but whether they should receive double lung transplantation (DLT) or single lung transplantation (SLT) is still controversial. The aim of this study was to determine which type of lung transplantation was more effective and relatively safe in IPF patients by meta-analysis.

**Methods:**

Publications comparing overall survival (OS) or other perioperative characteristics between IPF patients undergoing SLT and DLT were selected from electronic databases. The hazard ratios (HRs) were abstracted or calculated to evaluate the survival outcome. Odds ratios (ORs) or mean differences (MDs) were used to compare the causes of death or perioperative parameters. A random-effect model was used to combine data. Heterogeneity was quantified by means of an I^2^ with 95% confidence interval (95% CI). The publication bias was estimated using the Eggers test with Begg’s funnel plots.

**Results:**

16 studies with 17,872 IPF cases who met the inclusion criteria were included in this meta-analysis. SLT was associated with declined post-transplant FEV1% (MD = -15.37, 95% CI:-22.28,-8.47; *P*<0.001), FVC % (MD = -12.52, 95% CI:-19.45,-5.59; *P*<0.001) and DLCO% (MD = -13.85, 95% CI:-20.42,-7.29; *P*<0.001), but no significant advantage of DLT over SLT was seen in the overall survival outcome (HR = 1.08, 95% CI: 0.91–1.29; P = 0.391). Subgroup analyses for studies of follow-up period ≥ 60 months also showed similar results (all *P*-values>0.05). Moreover, there was fewer deaths attributable to primary graft dysfunction in SLT recipients (OR = 0.31, 95% CI: 0.2–0.48; P<0.001), while more patients with SLT died of malignancy (OR = 3.44, 95% CI: 2.06–5.77; P<0.001).

**Conclusion:**

Our findings suggest that DLT was associated with better postoperative pulmonary function, but there was no difference in long-term overall survival between patients undergoing DLT and SLT. However, further high-quality and large-scale studies are needed to confirm these findings.

## Introduction

Idiopathic interstitial pneumonia (IIP), with unknown etiology, has major implications for prognosis and management. Idiopathic pulmonary fibrosis (IPF), a chronic and progressive interstitial fibrotic lung disease, where no cause can be identified, is one of the most common types of IIP and remains a fatal disease with a median survival time from diagnosis of two to five years. Current treatment options for IPF are limited. Although antifibrotics, such as pirfenidone and nintedanib, have shown promising results in slowing disease progression [1, 2], it may bring potential serious side effects and help little for end-stage disease.

Lung transplantation remains the only curative treatment for end-stage lung disease showing a survival benefit and lower value on cost and procedural risk [3]. As an increasing number of IPF patients are present for lung transplantation, one question has arised which type of lung transplantation the patients should receive. This issue is particularly important since donor lungs suitable for transplantation are an scarce resource and should be appropriately allocated. For instance, if single lung transplantation (SLT) is considered non-inferior, double lung transplantation (DLT) may not be a prior choice. Unfortunately, the benefits of DLT versus single lung SLT is still controversial and debated in relevant literatures. Moreover, a prospective randomized trial is absent, and in that case, the lung transplant community can only rely on large registry analyses to illuminate this debate. Most recently, a large study with 4134 recipients demonstrated that DLT was associated with better graft survival than SLT in patients with IPF [4]. Later, numerous studies suggested that the use of SLT versus DLT in IPF did not correspond to significantly different survival [5–7].

Meta-analysis is an important tool to reliably and accurately summarize the current evidence. Although a recent meta-analysis has compared the two surgical procedures in end-stage lung diseases [8], it is still unclear which type of lung transplantation is more effective and relatively safe particularly in IPF patients, concerning post-transplant survival and main causes of death. Therefore, we conducted this study to pooled survival rate and overall survival between patients undergoing DLT and SLT. A key secondary objective was to assess the post-transplant parameters in IPF patients. We also investigated the main cause of death in two procedures to gain a better insight in lung transplantation in IPF.

## Materials and methods

We conducted this meta-analysis according to the Preferred Reporting Items for Systematic Reviews and Meta-Analyses (PRISMA) Statement protocol [9].

### Search strategy

Studies were identified via an electronic search of PubMed, Web of Science, Embase, and Cochrane Library (updated to October 1, 2019) by two investigators (DL and YL). The syntax used for search was ((lung transplantation) AND ((idiopathic pulmonary fibrosis) OR IPF)) AND (((prognosis) OR outcome) OR survival). The languages were limited to English. We also searched the reference lists in the initially identified articles to get additional relevant records and disagreements were resolved at each step by consensus. The full text of the included articles was examined to determine whether the articles contained relevant information.

### Selection criteria

The search results were screened according to the following inclusion criteria to ensure that studies selected were sufficient to test the hypotheses of the meta-analysis: (a) observational studies comparing SLT and DLT in patients with a diagnosis of IPF according to the guideline; (b) the association between type of procedure and overall survival (OS) in IPF had to be evaluated; (c) hazard ratios (HRs) with their 95% confidence intervals (95% CIs) for OS, odds ratios (ORs) or mean differences (MDs) with 95% CIs for perioperative parameters must be provided or could be calculated from the data presented. Reviews, conference abstracts, letter to editor or comments were excluded due to insufficient data. If more than one paper reported on the same study population, the most recent one was included. The Newcastle–Ottawa scale was applied to evaluate the quality of included studies, which allocates stars (maximum of 9) for quality of selection, comparability, exposure, and outcome of study participants. High quality studies were those with scores of five to nine, while those with scores of zero to four were considered low quality [10]. Two investigators (DL and YL) independently scored the quality of the studies using the same scale. Disagreements between the reviewers were resolved by consensus with a third author (BW).

### Data extraction

Two investigators (DL and YL) independently extracted data, blinded to the authors and institutions of the included studies. Discrepancies (if any) were addressed by joint re-evaluation of the original article. The following information was extracted from each study and used as a supplement if available: first author, year of publication, country, sample size, characteristics of the population (age, male%, mean pulmonary artery pressure), follow-up period, HR with 95% CI and data source. If HR with 95% CI could not be obtained directly, the data extracted through the Kaplan-Meier curves by GetDataGraph Digitizer 2.24 (http://getdata-graph-digitizer.com) was used to reconstruct the HR and its variance (GraphPad Software, Inc., La Jolla, CA, USA).

### Outcome assessment

The primary outcome measures were pooled 1-, 3-month and 1-, 3-, 5-year survival rates and pooled HRs. Secondary outcomes included postoperative pulmonary function, ventilator days, rates of extracorporeal membrane oxygenation (ECMO) and dialysis, hospital mortality, and postoperative hospital days.

### Statistical analysis

The HR with its 95% CI was abstracted or calculated to quantitatively evaluate the association between type of lung transplantation and IPF survival outcome using DerSimonian and Laird method. For secondary outcomes, the ORs with 95% CIs were used to compare the discontinuous parameters using Mantel Haenszel (MH) method, while MDs with 95% CIs were selected when comparing the continuous parameters that were reported in identical scales across all studies. Heterogeneity was quantified using a chi-squared test and by means of an I^2^ with 95% CI [11, 12]. A random-effect model was used to combine data [13, 14]. One-way sensitivity analysis was conducted to evaluate the stability of the results by deleting one study each time to reflect the influence of the individual data set to the pooled HR [15]. The publication bias was estimated using the Eggers test with Begg’s funnel plots [16]. For all analyses, a two-sided *P* value less than 0.05 was considered as statistically significant. All analyses were performed by Stata version 12.0 (Stata Corporation, College Station, TX) and Revman5.3 software (Cochrane Collaboration, Oxford, UK).

## Results

### Baseline characteristics of eligible studies

**Fig 1:**
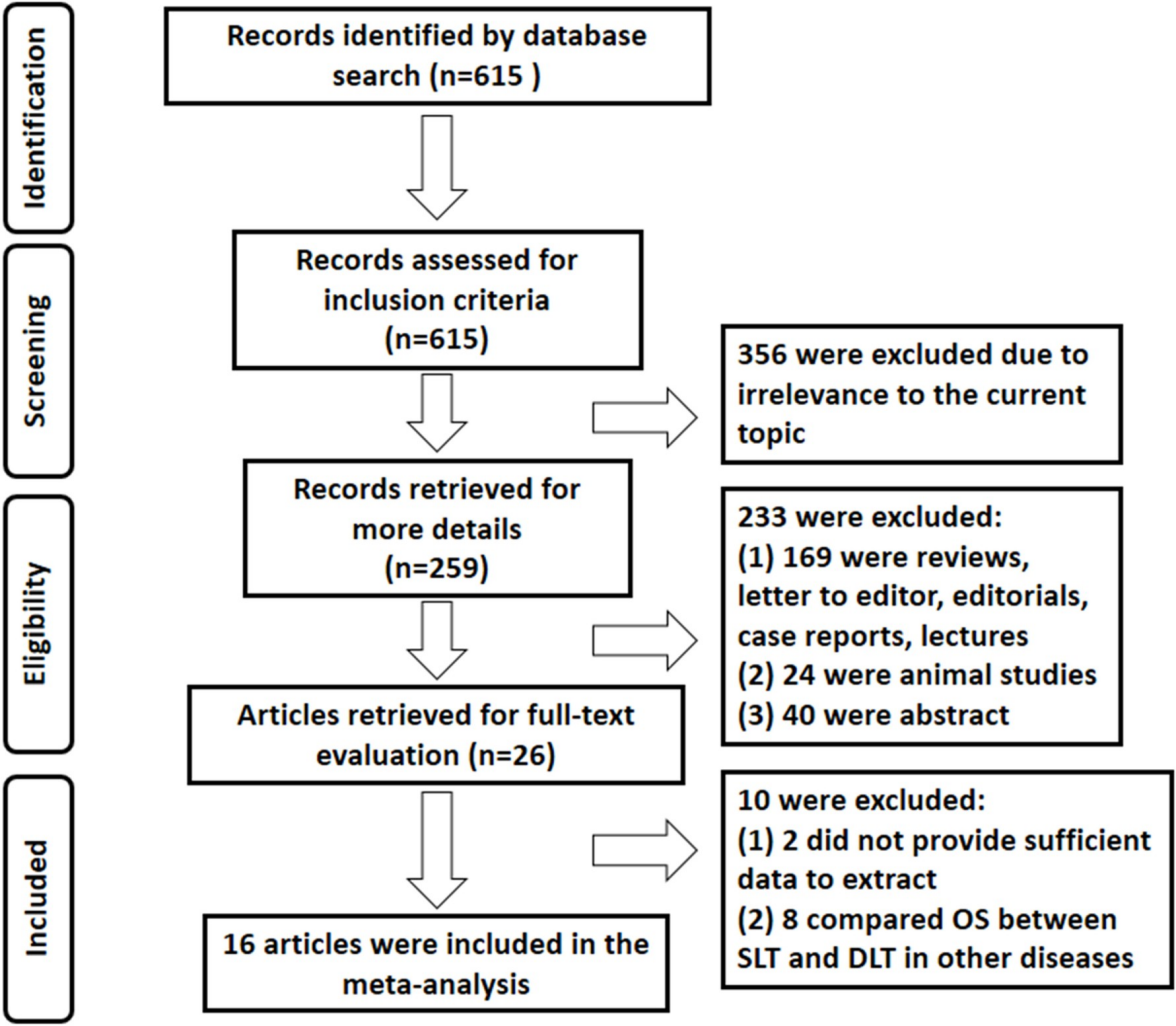
Flow chart of selection process for eligible articles.

A total of 615 related citations were identified based on the initial search. After independent review by inclusion criteria, 356 were excluded due to irrelevance to the current analysis, leaving 259 studies for retrieval of more details. Then, 233 studies were excluded since they were reviews, letters to editor, editorials, case reports, conference abstracts or animal studies. After detailed evaluation, 10 studies were excluded for lacking sufficient data to extract (n = 2), or the survival outcome evaluated in other diseases other than IPF (n = 8). Ultimately, 16 studies with 17,872 IPF cases distributed in five countries were included in this meta-analysis [4–7, 17–28] (Fig 1). These studies were published between 2005 and 2019, among which six were single-center studies, while the other ten were multi-center studies. All studies collected data retrospectively. HRs with 95% CIs were extracted directly from eight studies and calculated for the remaining eight. All the included studies were scored more than five and assessed as high-quality studies. The characteristics of these 16 studies included in the meta-analysis are presented in Table 1.

**Table 1:** Characteristics of eligible studies.

First author	Year	Country	Sample size	Follow-up months	Average age (years) (mean ± SD)	Male (%)	Mean PAP (mmHg) (mean±SD)	SLT (n)	DLT (n)	HR Estimate	NOS score
Meyer DM	2005	US	821	36	53.6 ± 8.1	NA	25.4	636	185	Curve	6
Nwakanma LU	2007	US	429	60	62.7 ± 2.4	58.5	NA	349	80	Curve	6
Mason DP	2007	US	82	72	52 ± 11	63	27 ± 12	50	32	Curve	7
Weiss ES	2009	US	1256	12	57.6 ± 9.3	68.8	25.9	655	601	HR	6
Thabut G	2009	US	3327	60	56 ± 9.4	68	25.2	2146	1181	HR	8
Neurohr C	2010	Germany	76	60	52.4 ± 1.2	56.6	27.9 ± 1.2	46	30	HR	8
Force SD	2011	US	3860	240	54.8 ± 10.5	65.9	25.7 ± 10.2	2431	1429	HR	7
Wang Q	2011	UK	257	144	52.6 ± 8.9	66.5	NA	195	62	HR	8
De Oliveira NC	2012	US	79	120	NA	NA	NA	65	14	Curve	7
Lehmann S	2014	Germany	58	72	54 ± 10	69	NA	39	19	Curve	7
Schaffer JM	2015	US	4134	60	60 ± 8.2	72	25.6 ± 9.3	2010	2124	HR	8
ten Klooster L	2015	Netherlands	52	120	53 ± 8	74	23 ± 10	21	31	HR	7
Chauhan D	2016	US	1002	84	NA	NA	NA	434	568	Curve	6
Ranganath NK	2019	US	2179	120	62.4	72.5	NA	974	1205	Curve	7
Wei D	2019	China	109	24	60.1 ± 9.1	92.7	33.8 ± 14.7	70	39	Curve	5
Spratt JR	2019	US	151	60	58.6 ± 7.9	62.9	25.2 ± 8.4	94	57	HR	8

DLT, double lung transplantation; HR, hazard ratio; CI, confidence interval; NA, not available; NOS, Newcastle–Ottawa scale; PAP, pulmonary artery pressure; SLT, single lung transplantation.

### Survival of IPF patients

Totally, 10,215 (57.2%) recipients underwent SLT and 7,657 (42.8%) recipients underwent DLT. Several studies independently reported 1-month, 3-month, 1-year, 3-year and 5-year survival statistics, therefore we performed meta-analysis on the pooled survival rate. The pooled 1-, 3-month and 1-, 3-, 5-year survival rates for SLT and DLT were 87.4 vs 91.7, 84.3 vs 88.9, 78.4 vs 79.6, 50.7 vs 52, 54.9 vs 51.1, respectively.

**Fig 2:**
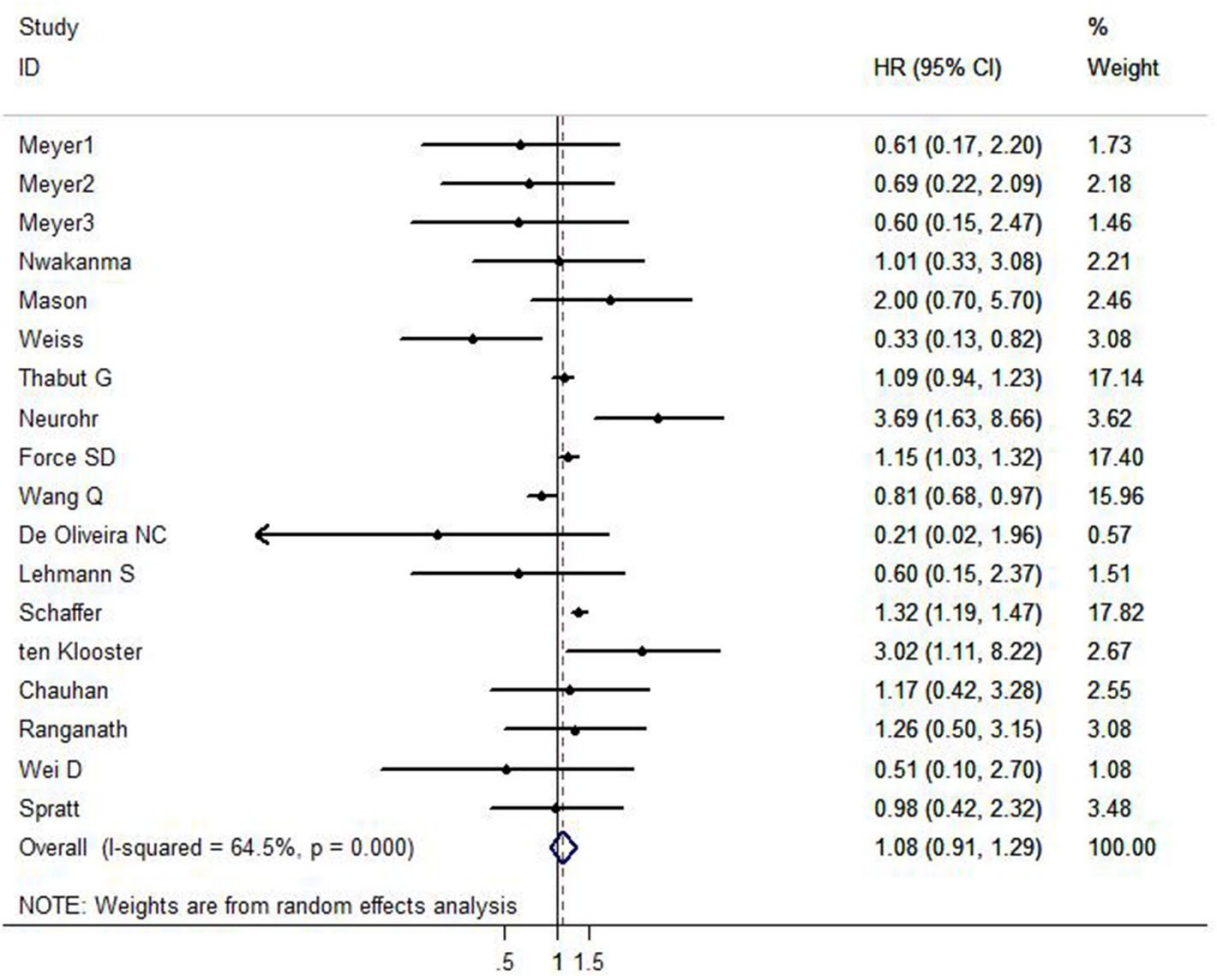
Overall survival between recipients undergoing DLT versus SLT stratified by HR estimation. The summary HR and 95% CIs were shown (according to the random effect estimations).

The pooled HRs of this meta-analysis are summarized in Table 2. All of the studies had a maximum follow-up period exceeding 1 year. Four of the sixteen included studies suggested that outcomes for SLT for patients with IPF were superior to those for DLT, while the other four studies showed DLT was associated with better survival than SLT. The remaining ten reported that there was no statistical difference in OS between recipients undergoing DLT versus SLT, implying controversial results among current studies. Survival was compared by procedure type within three age groups in Meyer’s study. Therefore, in this meta-analysis, analysis in each age group was regarded as an independent study. By pooling the data using a random-effect model, as shown in Fig 2, the results suggested that the OS for IPF patients undergoing SLT was lower than those with DLT, but this did not reach statistical significance (HR = 1.08, 95% CI: 0.91–1.29; *P* = 0.391), indicating that the use of SLT versus DLT in IPF did not correspond to significantly different survival.

**Table 2:** Meta-analysis of the overall survival of IPF patients undergoing SLT versus DLT.

Subgroups	Number of studies	HR (95% CI)	*P*-value	I^2^ (%) (95% CI)	*P*_*het*_
Total	18	1.08 (0.91–1.29)	0.391	64.5 (41.4–78.5)	<0.001
*Follow-up period*					
<60 months	5	0.50 (0.29–0.85)	0.011	0 (0–79.2)	0.869
60 months	5	1.27 (1.01–1.59)	0.028	66.2 (12–87)	0.019
60–120 months	3	1.24 (0.65–2.37)	0.516	0 (0–89.6)	0.392
120 months	3	1.32 (0.43–4.05)	0.625	58.5 (0–88.2)	0.090
>120 months	2	0.97 (0.69–1.37)	0.866	90.1 (65.9–97.4)	0.002
*Age group*					
≥60 years	3	0.63 (0.26–1.49)	0.292	11.7 (0–52.7)	0.322
<60 years	3	1.24 (0.36–4.32)	0.738	75.5 (19.2–92.6)	0.017
*Publication date*					
Before 2014	11	1.00 (0.80–1.26)	0.987	67.2 (38.3–82.6)	0.001
After 2014	7	1.31 (1.19–1.46)	<0.001	0 (0–70.8)	0.46
*Sample size*					
≥1000	6	1.15 (0.99–1.34)	0.065	61.8 (6.9–84.3)	0.023
<1000	12	1.06 (0.71–1.60)	0.767	53.1 (9.7–75.6)	0.015
*Data source*					
multi-center	12	1.05 (0.88–1.25)	0.584	68.8 (43.2–82.8)	<0.001
single-center	6	1.15 (0.54–2.44)	0.723	57.7 (0–82.9)	0.038

DLT, double lung transplantation; HR, hazard ratio; CI, confidence interval; *P*_*het*_, *P*-value for heterogeneity; SLT, single lung transplantation.

There was obvious heterogeneity among studies (*P*_*het*_ <0.001 and I ^2^ = 64.5%), so we performed subgroup analyses according to confounders, such as follow-up period (<60 months, 60 months, 60–120 months, 120 months or >120 months), age group (≥60 years or <60 years), sample size (≥1000 or <1000), publication date (before 2014 or after 2014) and data source (multi-center or single-center). In the subgroup analysis based on the follow-up period, the combined HR was 0.50 (95% CI: 0.29–0.85) for studies of follow-up period <60 months and 1.27 (95% CI: 1.01–1.59) for those of 60-month follow-up period. However, for studies of follow-up period ≥ 60 months, HRs did not differ significantly between the two groups. Furthermore, the summary HR of the studies published within 5 years (after 2014) was 1.31 (95% CI: 1.19–1.46), indicating lower OS for IPF patients undergoing SLT, as shown in Table 2.

### Postoperative parameters of IPF patients

Several studies independently evaluated postoperative parameters including pulmonary function (FEV1%, FVC % and DLCO %) [7, 22], ventilator days [26, 28], rates of ECMO [25, 26] and dialysis [25, 26], hospital mortality [25, 26], and postoperative hospital days [7, 28]. Our analyses suggested that SLT was associated with declined post-transplant FEV1% (MD = -15.37, 95% CI: -22.28, -8.47; *P*<0.001), FVC % (MD = -12.52, 95% CI: -19.45, -5.59; *P*<0.001) and DLCO % (MD = -13.85, 95% CI: -19.15, -8.56; *P*<0.001) (Table 3), while no significant differences were observed in postoperative ventilator days, ECMO support, dialysis, hospital mortality, or hospital day between the two groups.

**Table 3:** Comparison of postoperative parameters between SLT and DLT in patients with IPF.

Clinical parameters	Number of studies	Number of patients		OR (95% CI)	MD (95% CI)	*P*-value	I^2^ (%) (95% CI)	*P*_*het*_
		SLT	DLT					
FEV1%	2	81	42	-	-15.37 (-22.28,-8.47)	<0.001	0 (0–99.9)	0.92
FVC %	2	81	42	-	-12.52 (-19.45,-5.59)	<0.001	0 (0–99.9)	0.79
DLCO %	2	81	42	-	-13.85 (-20.42,-7.29)	<0.001	35 (0–78.8)	0.22
Ventilator days	2	133	76	-	-0.26 (-14.48, 13.95)	0.97	83 (30–96)	0.01
ECMO support	2	120	49	0.64 (0.12, 3.45)	-	0.61	28 (0–72.7)	0.24
Dialysis	2	120	49	0.24 (0.05, 1.06)	-	0.06	0 (0–99.9)	0.75
Hospital mortality	2	120	49	0.62 (0.24, 1.56)	-	0.31	0 (0–99.9)	0.73
Length of stay	2	173	87	-	4.49 (-18.42, 27.40)	0.70	92 (70.6–97.6)	<0.001

DLCO, carbon monoxide diffusion capacity; DLT, double lung transplantation; ECMO, extracorporeal membrane oxygenation; FEV1, force expiratory volume in 1 second; FVC, forced vital capacity; OR, odds ratio; MD, mean difference; CI, confidence interval; *P*_*het*_, *P*-value for heterogeneity; SLT, single lung transplantation.

### Cause of death in IPF patients

Meta-analysis of the main causes of death by type of lung transplantation is depicted in Table 4. There were a significantly higher number of deaths attributable to primary graft dysfunction in DLT recipients (*P*<0.001). Among patients who had SLT, 148 of 1190 (12.4%) died of malignancy, compared with 16 of 426 (3.8%) of those who had DLT (*P*<0.001).

**Table 4:** Meta-analysis of the main causes of death in SLT and DLT.

Cause of death	Number of studies	Number of patients (Event/Total)		OR (95% CI)	*P*-value	I^2^ (%) (95% CI)	*P*_*het*_
		SLT	DLT				
Infection	4	305/1216	100/433	1.15 (0.86–1.55)	0.35	1 (0–11.9)	0.39
Primary graft dysfunction	3	47/1195	47/424	0.31 (0.2–0.48)	<0.001	0 (0–89.6)	0.48
Malignancy	3	148/1190	16/426	3.44 (2.06–5.77)	<0.001	0 (0–89.6)	0.86

DLT, double lung transplantation; OR, odds ratio; CI, confidence interval; *P*_*het*_, *P*-value for heterogeneity; SLT, single lung transplantation.

### Publication bias and sensitivity analyses

**Fig 3:**
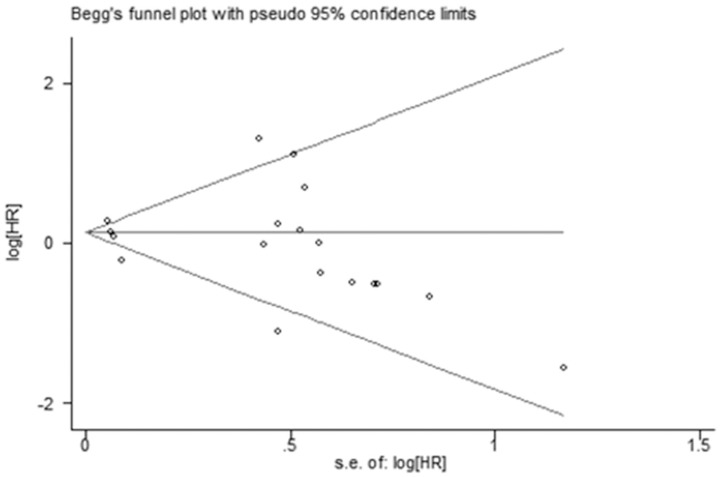
Egger’s plot to detect publication bias on overall estimate.

Publication bias in current meta-analysis was tested by Egger’s test with Begg’s funnel plots. The shape of the funnel plot of the pooled HR did not reveal any evidence of asymmetry, with *P* = 0.399 in the Egger’s test (Fig 3). For sensitivity analyses, all the results were not materially altered by omitting one study per time.

## Discussion

The primary goal of lung transplantation is to provide a survival benefit for patients with chronic end-stage lung disorders, including IPF. Results from current published articles have implied that there might be a preference to perform DLT for interstitial lung diseases with better survival rates, especially in high-risk patients, young patients, and patients with high mPAP [27, 29]. Recently, Villavicencio et al retrospectively analyzed recipients of lung transplants for pulmonary fibrosis between 1987 to 2015, and found that DLT improved survival compared with SLT, which should be considered the procedure of choice in patients younger than 70 years old [30]. Nevertheless, shorter procedure time, less operative trauma, and avoidance of longer ischemic time for the second lung with the risk of organ failure are arguments favoring SLT [17]. Thus, for at least a certain subgroup of patients, SLT is still an appropriate therapy. Rather, some recent studies reported no superior survival for DLT over SLT for IPF recipients. Thabut et al analyzed the United Network for Organ Sharing (UNOS) data of 3,327 IPF patients who underwent transplantation between 1987 and 2009, and found that there was no difference in long-term survival between SLT and DLT patients after risk adjustment by multivariable regression and propensity-based matching [21]. In the present meta-analysis, we summarized the results of 16 clinical studies representing 17,872 IPF patients. By pooling all the studies and comparing the survival outcome of patients according to the type of lung transplantation, we observed that patients undergoing DLT had higher 1-, 3-month and 1-, 3-year survival rates, and better overall survival than those receiving SLT, but this did not reach statistical significance. These results are consistent with previous studies showing equivalent survival between SLT and DLT for IPF recipients.

An interesting phenomenon is that the mixed results even occurred among enrolled studies that analyzed different patient subsets using similar, temporally overlapping large registry data, indicating possible selection biases. Besides, most of the included studies were retrospective and differed in their study designs, and population characteristics. Therefore, to minimize the heterogeneity, we conducted subgroup analyses stratified by confounders. As a result, equivalent OS between SLT and DLT was also proved when stratified by age, sample size and data source. However, we found that patients undergoing SLT had better OS for studies with shorter follow-up period (<60 months), while the pooling results for studies with longer follow-up periods (>60 months) showed no difference in survival between SLT and DLT group, implying that the long-term prognosis is similar for IPF patients treated with these two procedures. Notably, OS was lower for patients undergoing SLT by pooling studies published within 5 years (after 2014).

Despite no significant advantage of DLT over SLT in survival outcome, DLT group showed better postoperative pulmonary function as reflected by FEV1%, FVC % and DLCO %. Reasons for the improved lung function with DLT are likely associated with enhanced pulmonary reserve and improved respiratory mechanics. Nevertheless, recipients of either DLT or SLT experienced similar period of mechanical ventilation, ECMO support, overall hospital days, as well as hospital mortality. This discrepancy may be explained by continual improvements in postoperative care and by improved surgical strategies.

Postoperative mortality after lung transplantation is mainly caused by various complications. As expected, primary graft dysfunction was more frequent as a cause of death after DLT rather than SLT (11.1% vs. 3.9%; P <0.001), which could explain the increased early mortality that other previous investigators observed after DLT. We also found a higher incidence of cancer after SLT than after DLT, which might be attributed to complications involving the remaining lung in cases of SLT. In Wei’s study, malignant tumors were only seen in SLT recipients [7]. Magruder et al also indicated that SLT was independently associated with de novo malignancy [31]. In addition, a higher infection rate was observed in SLT group, though this difference was not apparent. It should be mentioned that SLT remained a significant predictor for bronchiolitis obliterans syndrome (BOS) stage ≥1 and death [22]. As BOS is associated with recurrent viral, bacterial and fungal infections, and fatal infectious complications in SLT patients has been increasing, the impact of the native lung and different pathogens remains to be elucidated in studies with a larger sample size.

Some methodological limitations of current meta-analysis were inevitable and should be taken into consideration when interpreting the results. First of all, some clinical indicators were only evaluated in a few studies, thus weakening the effectiveness of meta-analysis. For instance, postoperative complications and causes of death were analyzed by pooling two or three studies. Some important operation-related complications like chronic rejection and respiratory failure were not assessed as only one article had sufficient data. Second, lack of the original data of enrolled studies limited our further analysis to determine the optimal surgical treatment for certain subgroup of patients with IPF. For example, Villavicencio et al found that among SLT recipients, patients with a pulmonary artery pressure ≥30 mm Hg and an allocation score ≥45 had decreased survival, while pulmonary artery pressure and allocation score did not affect survival in DLT recipients [30]. Also, although registry data of different time periods were analyzed in different studies, lack of the original data at a patient level also limited our ability to exclude possible duplicates from multi-center studies. Therefore, subgroup analyses based on single-center studies were conducted to overcome this limitation. Third, selection bias in some studies may affect the judgement on an age cutoff at which DLT should be avoided. Given the shortage of donor lungs availability, it is crucial to understand whether there is a subgroup of IPF patients for whom DLT does offer a true survival advantage [32]. Fourth, although we addressed the heterogeneity by using a random-effects model along with prespecified subgroup and sensitivity analyses, our study was based on published literature, which limited our ability to correct for potential confounding factors if they were not reported or not using unified data [33]. Particularly, high levels of undetected heterogeneity may exist in very small meta-analyses [34]. Thus, pooled results of small subgroup analyses should be interpreted with caution and appropriate multivariate analysis will be important in future studies to examine which type of operation can improve the outcomes, independent of other known pretransplant factors such as age, sex, disease severity, treatment, and donors’ characteristics. Fifth, the exclusion of unpublished papers, abstracts and letters to the editor may lead to potential publication and reporting bias, since positive results are more likely to be acceptable by journals. Due to the fact that only a few studies meeting inclusion criteriwere included, it is possible that some other reports may negatively influence the present results. Finally, some survival outcomes were calculated from Kaplan-Meier curves, which may have introduced some imprecision.

Despite these limitations and some methodological imperfections, this study was based on retrospective data from large patient registries and single-center cohorts, which offered evidence for decision-making in a real-world environment. Nonetheless, patients undergoing SLT and DLT may be differed with regard to some pre-transplant recipient-related factors. Careful consideration of patient factors should weigh into the decision.

In conclusion, the present study comprehensively evaluated the prognostic performance of SLT and DLT in IPF recipients. Our results demonstrated that DLT was associated with better postoperative pulmonary function, but there was no difference in long-term overall survival between patients undergoing DLT and SLT. However, further high-quality and large-scale studies are needed to confirm these findings with attention to multiple perioperative factors and the context of the individual patient’s risk profile.

## Supporting information

S1 Table Agreement test for Newcastle–Ottawa scale scores evaluated by reviewers. (DOCX)

S1 Checklist PRISMA checklist. (DOC)
