# Supplementary material for: Single versus bilateral lung transplantation in idiopathic pulmonary fibrosis: A systematic review and meta-analysis
Source: PLoS One. 2020 May 21;15(5):e0233732. doi: 10.1371/journal.pone.0233732 (PMC7241801; doi:10.1371/journal.pone.0233732)
Supplement: S1 Table — (DOCX) [file pone.0233732.s001.docx]

**Table S1. Agreement test for Newcastle–Ottawa Scale scores evaluated by reviewers**

| Study | NOS1 | NOS2 | NOS3 |
| --- | --- | --- | --- |
| Meyer DM | 6 | 6 |  |
| Nwakanma LU | 6 | 6 |  |
| Mason DP | 7 | 7 |  |
| Weiss ES | 6 | 7 | 6 |
| Thabut G | 8 | 8 |  |
| Neurohr C | 8 | 8 |  |
| Force SD | 8 | 7 |  |
| Wang Q | 8 | 8 |  |
| De Oliveira NC | 7 | 7 |  |
| Lehmann S | 7 | 7 |  |
| Schaffer JM | 8 | 8 |  |
| ten Klooster L | 7 | 7 |  |
| Chauhan D | 6 | 6 |  |
| Ranganath NK | 7 | 7 |  |
| Wei D | 6 | 5 | 5 |
| Spratt JR | 8 | 8 |  |

NOS = Newcastle–Ottawa Scale; NOS1=NOS score evaluated by reviewer1; NOS2=NOS score evaluated by reviewer2; NOS3=Disagreements between the reviewers were resolved by consensus with the third author.
